# Choroidal osteoma: a rare clinical image

**DOI:** 10.11604/pamj.2023.44.48.38696

**Published:** 2023-01-25

**Authors:** Manal Tabchi, Zeinabou Hmeimett

**Affiliations:** 1 Ophthalmology Department A, Hospital of Specialties in Rabat, Rabat, Morocco

**Keywords:** Choroidal osteoma, choroidal neovascularization, bone tumor

## Image in medicine

Choroidal osteoma is a rare benign bone tumor. It is unilateral in two-thirds of cases. It occurs unilaterally and more often in young women. The tumor is most often located near the papilla. The complications classically described are progressive retinal atrophy and choroidal neovascularization. We report the case of a 39-year-old female patient, with no previous history, who consulted us for a sudden unilateral decrease in visual acuity, associated with metamorphopsias and a para-central scotoma. Fundus examination of the right eye revealed a large, yellowish-white choroidal tumor with fairly well-delineated margins in a juxta-papillary arrangement. Fluorescein angiography showed an early hyperfluorescence becoming intense at late times, associated with papillary edema. In front of this picture, we evoke the hypothesis of a choroidal neovascular membrane. The optical coherence tomography (OCT) showed the presence of a hyper-reflective pre-epithelial lesion in the inter-papillomacular region, associated with a retinal serous detachment, indicating a choroidal neovascular complication. B-mode ultrasonography confirmed the diagnosis by showing a hyperechoic thickening of the choroid with a posterior shadow cone. In our case, it was a choroidal osteoma complicated by optic neuropathy and choroidal neovascularization. The patient received several intravitreal injections of anti-vascular endothelial growth factor (anti-VEGF), followed by long-term monitoring. Choroidal osteoma is a rare benign tumor. It often progresses slowly, but may have complications, in particular choroidal neovascularization, the treatment of which is not well codified. The long-term visual acuity is often very poor.

**Figure 1:**
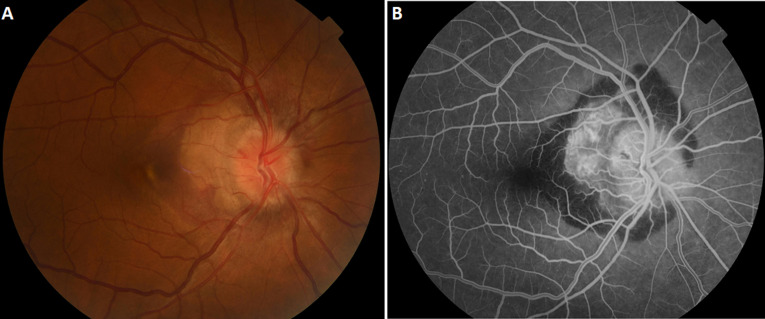
A) retinography of the right eye showing a choroidal osteoma; B) fluorescein angiography of the right eye showing intense hyperfluorescence, and papillary edema

